# Timoshenko beam model for buckling of piezoelectric nanowires with surface effects

**DOI:** 10.1186/1556-276X-7-201

**Published:** 2012-03-27

**Authors:** Arash Tourki Samaei, Majid Bakhtiari, Gang-Feng Wang

**Affiliations:** 1 Young Researchers Club, Chalous Branch, Islamic Azad University, Chalous, 46619/61367, Iran; 2 School of Mechanical Engineering, Iran University of Science and Technology, Narmak, Tehran, 16846, Iran; 3 Department of Engineering Mechanics, SV Laboratory, Xi'an Jiaotong University, Xi'an, 710049, People's Republic of China

**Keywords:** surface elasticity, buckling, piezoelectric nanowire

## Abstract

This paper investigates the buckling behavior of piezoelectric nanowires under distributed transverse loading, within the framework of the Timoshenko beam theory, and in the presence of surface effects. Analytical relations are given for the critical force of axial buckling of nanowires by accounting for the effects of surface elasticity, residual surface tension, and transverse shear deformation. Through an example, it is shown that the critical electric potential of buckling depends on both the surface stresses and piezoelectricity. This study may be helpful in the characterization of the mechanical properties of nanowires and in the calibration of the nanowire-based force sensors.

## Introduction

Nanowires have attracted considerable attention in the literature for future applications as sensors, actuators, transistors, and resonators in nanoelectromechanical systems and in biotechnology [1]. Because of these varied applications, it is very important to accurately characterize the mechanical properties of nanowires and their response to external loading. In atomistic scales, owing to the increasing ratio of surface area to volume, the stress and strain effects on surface physics become very important [2]. In this regard, theoretical and experimental investigations have provided a better understanding of the effects of stress on surface physics [3,4]. For example, by conducting bending tests using atomic force microscopy, Cuenot et al. [4] have demonstrated that the stiffness of nanowires is size-dependent, and this phenomenon has been theoretically explained by considering the surface effects [5-8]. He and Lilley [6] investigated the influences of surface tension on the static bending of nanowires. Wang and Feng [8] studied the surface effects on the buckling and vibration behaviors of nanowires, based on the Laplace-Young equation. The theoretical investigations related to the surface effects and mechanical behavior show a good agreement with the experiments and atomistic simulations [3,6,9].

Recently, piezoelectric nanostructures, such as nanowires, have been drawing a lot of attention due to their potential applications as nanoresonators [10], diodes [11], and nanogenerators [12]. Piezoelectric nanomaterials exhibit size-dependent properties at nanoscale, and also, it has been demonstrated that they have larger piezoelectric constants than their bulk counterparts [13,14]. Experimental measurements and atomistic simulations demonstrate that the elastic and fracture properties of ZnO piezoelectric nanowires vary with their cross-sectional dimensions [5,15,16]. Zhao et al. [17] found out that the effective piezoelectric coefficient of the ZnO nanowire is frequency-dependent and that it is much larger than that of the bulk material. Using the perturbation theory [13] and finite element method [14], the electrostatic potential in a bending piezoelectric nanowire was calculated. For the first time, Wang and Feng [18] used the Euler-Bernoulli beam model to investigate the buckling and vibration behaviors of piezoelectric nanowires by taking into account the effects of surface stresses and piezoelectricity. Also, surface effects and surface piezoelectricity are considered to study the electromechanical coupling behavior of piezoelectric nanowires with the Euler beam theory by Yan and Jiang [19].

The objective of the present paper is to investigate the combined surface and piezoelectric effects on the buckling of piezoelectric nanowires using the modified Timoshenko beam model. In this study, the two modified Euler beam and Timoshenko beam models have been compared, but no quantitative experimental measurement has been reported on the buckling condition of piezoelectric nanowires. A numerical example is presented in the article to demonstrate both the surface and piezoelectric effects, and then, some discussions are provided based on the obtained results.

## Formulation of the problem

**Figure 1:**
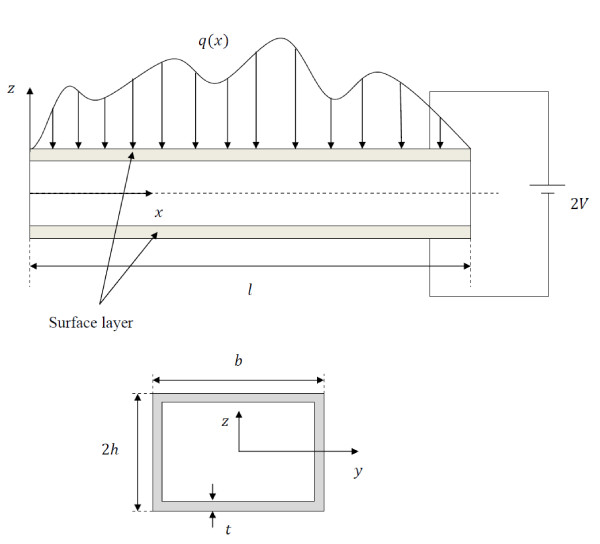
**Schematic of a piezoelectric nanowire with surface layers**.

The problem envisaged in this article is a hinged-hinged piezoelectric nanowire with length *l*, width *b*, and height 2*h*, as shown in Figure 1. The mechanical properties of the bulk part (*E*, *G*, and *ρ*) designate the Young's modulus, shear modulus, and mass density of the nanowire, respectively.

In the current study, a crystalline ZnO nanowire with the C_6v _symmetry about the poling direction along the *z*-axis [18] is considered, which has a surface layer with surface elasticity modulus (*E*^5^) [20-22], which can be determined by atomistic simulations or experiments [3,23], surface layer thickness (*t*) [8], and constant residual surface tension (*τ*^0^) [18,24]. The effect of the residual surface stress acting as a transverse load on the nanowire is calculated by the Laplace-Young equations [25].

The ratio of surface energy *γ*(*J*/*m*^2^) to Young's modulus *E*(*J*/*m*^3^), *γ*/*E*, leads to some intrinsic length scale material parameter in the nanometer range [26,27]. When a material element has a characteristic length comparable to the intrinsic scale, the surface/interface energy can play an important role in its properties and behavior. According to Gibbs [26], the surface stress tensor $\sigma_{\alpha \beta }^{\text{5}}$ is related to the surface energy density *γ *as follows:

(1) $$\sigma_{\alpha \beta }^{\text{5}}=\gamma \delta_{\alpha \beta }+\partial \gamma /\partial \varepsilon_{\alpha \beta }^{\text{5}}.$$

where $\varepsilon_{\alpha \beta }^{\text{5}}$ and $\delta_{\alpha \beta }$ represent the surface strain tensor and the Kronecker delta, respectively. A one-dimensional and linear surface constitutive equation of Equation 1 is stated by introducing a set of surface elastic constants [4]:

(2) $$\sigma^{5}=\tau^{0}+E^{5}\varepsilon .$$

Equations 1 and 2 declare that the elastic responses of nanoscale elements largely depend on the surface elastic constants, which could be obtained either by atomistic simulations or experimental measurements [4,28]. In the present paper, the beam aspect ratio (aspect ratio corresponds to length-to-height ratio) is relatively small; the thick beam model needs to be applied to take the shear deformations into consideration. Therefore, based on the Timoshenko beam theory, the Laplace-Young equation predicts the transverse load on the nanowire as follows [8]:

(3) $$q\left(x\right)=H\frac{\partial^{2}w}{\partial x^{2}}\;H=2\tau^{0}b$$

The curvature of a bending beam is approximated by ∂^2^*w*/∂*x*^2^, where *w *is the deflection at the position *x *[29]. For the current study, the electric field is assumed to exist in the *z *direction. For the one-dimensional piezoelectric nanowire, the strain and stress can be obtained as follows [30]:

(4) $$\varepsilon_{x}=-z\frac{\partial^{2}w}{\partial x^{2}}\;\sigma_{x}=c_{22}\varepsilon_{x}-e_{32}E_{z}\;E_{z}=\frac{\partial \psi }{\partial_{z}}\;\tau =G\gamma^{0},$$

where *c*_22_, *e*_32_, and *ψ *are the linear elastic constant, the linear piezoelectric coefficient, and the electric potential of the piezoelectric material, respectively, and $\gamma^{0}=\frac{\partial w}{\partial x}-\phi$ is the shear strain. For the piezoelectric nanowire, the electric displacements with the applied strain and electric field for the piezoelectric nanowire can be written as follows [30]:

(5) $$D_{x}=\lambda_{11}E_{x}\;D_{z}=e_{31}\varepsilon_{x}+\lambda_{33}E_{z}\;E_{x}=-\frac{\partial \psi }{\partial x},$$

where *λ*_11 _and *λ*_33 _are the dielectric constants. The electric field component is *E_x _*≪ *E_z _*because the electric potential is almost constant along the nanowire (*x*-axis) except around the two ends [13]. Using the electrostatic equilibrium condition, the electric potential can be obtained [18], and thus, the stress of the piezoelectric nanowire can be calculated. For the hinged-hinged piezoelectric nanowire, a resultant axial force (*T_x_*) is induced by the applied electric potential as follows [18]:

(6) $$T_{x}=b\int_{-h}^{h}\sigma_{x}dz$$

The kinematic energy of the system includes the piezoelectricity effect and the residual surface tension acting as an external distributed load on the piezoelectric nanowire, whose work should be calculated. The energy method was employed to obtain the differential equation of the Timoshenko beam by considering the effects of both the surface elasticity and piezoelectricity. To derive the governing equation for the piezoelectric nanowire, considering the surface effects, the present study has followed the same procedure as that in the work of Rao [29] for an elastic beam. Also, the influence of surface stresses is modeled as a curvature-dependent transverse loading, and the piezoelectric effect induces an axial force in the piezoelectric nanowire. Considering these two mechanisms, the equilibrium equation of the beam is obtained [29] as follows:

(7) $$\left(1+\frac{\tau^{0}+Ve_{31}}{\kappa Gh}\right)\left(c_{11}\frac{2bh^{3}}{3}+\frac{e_{31}^{2}}{\lambda_{33}}\frac{2bh^{3}}{3}\right)\frac{\partial^{4}w}{\partial x^{4}}-2b\left(\tau^{0}+Ve_{31}\right)\frac{\partial^{2}w}{\partial x^{2}}=0,$$

where *A *and *κ *are the cross-sectional area and the shear correction factor of the cross section, respectively. Generally, the shear correction factor is considered to be in the range of 0.833 ≤ *κ*^2 ^≤ 0.870. Since the applied electric potential may induce a compressive axial force due to piezoelectric effects, it is essential to determine the critical electric potential for the buckling of piezoelectric nanowires. Solving Equation 7 for the hinged-hinged nanowire, the critical electric potential corresponding to the buckling of the noted nanowire can be obtained as follows:

(8) $$2b\left(\tau^{0}+e_{31}V\right)=-\frac{\xi {\left(\frac{\pi }{l}\right)}^{2}\left(c_{11}\frac{2bh^{3}}{3}+\frac{e_{31}^{2}2bh^{3}}{\lambda_{33}\;3}\right)}{1+\xi {\left(\frac{\pi }{l}\right)}^{2}\left(\frac{\left(c_{11}\frac{2bh^{3}}{3}+\frac{e_{31}^{2}2bh^{3}}{\lambda_{33}\;3}\right)}{\kappa GA}\right)},$$

where *ξ *is the slenderness ratio (slenderness ratio corresponds to length-to-the least radius of gyration of the cross section ratio) of the nanowire, and for a hinged-hinged beam, *ξ *= 1 [8]. Equation 8 presents a relation between the residual surface stress of a piezoelectric nanowire and the critical electric potential in the buckling analysis. Therefore, it can be inferred that the elasticity modulus and the residual surface stress could be obtained by measuring the critical electric potentials of two nanowires with different sizes [18].

## Example and discussion

To demonstrate the effects of both the surface elasticity and piezoelectricity on the buckling of a piezoelectric nanowire, a crystalline ZnO nanowire is considered for a case study. The bulk material property constants of this nanowire are the following: *c*_11 _= 207 Gpa, *e*_31 _= -0.51 C/m^2^, and *λ*_33 _= -7.88 × 10^-11 ^F/m [18], and its surface energy density on the (0001)-plane is *γ *= 1.6 J/m^2 ^[30]. The formulations in the 'Formulation of the problem' section indicate that the buckling behavior of piezoelectric nanowires largely depends on their surface and piezoelectric properties, which could be determined either by experiments or atomistic simulations [3,6,28,30]. For example, Miller and Shenoy [3] determined the free surface properties of aluminum using the embedded atom method for some crystallographic directions. By neglecting the shear deformation effect in Equation 8, the Euler model with surface effects is obtained [18] as follows:

(9) $$2b\left(\tau^{0}+e_{31}V\right)=-{\left(\frac{\pi }{l}\right)}^{2}\left(c_{11}\frac{2bh^{3}}{3}+\frac{e_{31}^{2}}{\lambda_{33}}\frac{2bh^{3}}{3}\right)$$

It is found from Equation 8 that the shear deformation lowers the critical compression force of buckling in comparison with the classical Euler solution.

**Figure 2:**
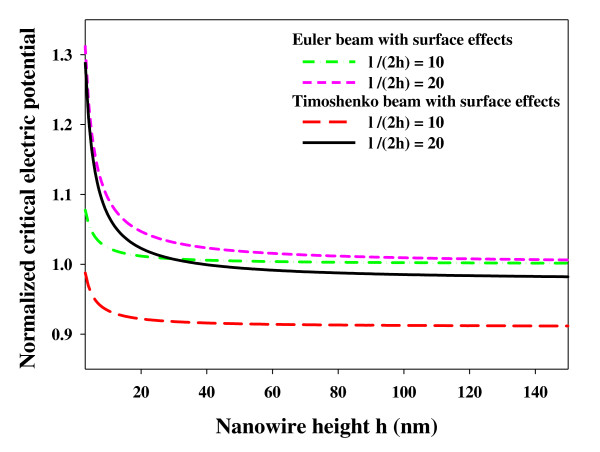
**Influence of surface effects, shear deformation, and piezoelectricity on normalized critical electric potential of the piezoelectric nanowire**.

The distributed shear loading arising from the surface tensions and axial tension which are produced by the electric potential could cause buckling in nanowires. To determine the buckling behavior of piezoelectric nanowires, the governing equation of motion (shown in the 'Formulation of the problem' section) has been obtained based on the surface and piezoelectric effects, which shows the significant influence of these effects. To better demonstrate these effects on the buckling behavior of the nanowire, the normalized critical electric potential (*Vcr*/*Vcr*0) has been plotted versus the nanowire height (*h*) in Figure 2. Here, *Vcr*0 is the critical electric potential for the buckling obtained from the Euler model without the surface effects. Figure 2 has been plotted for the first mode of buckling and also for several aspect ratios. By observing the curves, it can be concluded that, contrary to the classical model, the normalized critical electric potential depends on a size characteristic such as the nanowire height. These curves show that by reducing the nanowire height to the sub-20-nm range, both the surface and piezoelectric effects become more and more influential. Moreover, as the nanowire height increases, the curves converge and ultimately turn into those of the classical case, pointing out the fact that with the increase of the nanowire height, the surface effects are eliminated. On the other hand, it is seen that the normalized critical electric potential obtained from the modified Timoshenko beam model is smaller than that obtained from the modified Euler beam model, which shows that, in order to get more exact results, both the surface and shear deformation effects should be taken into consideration.

**Figure 3:**
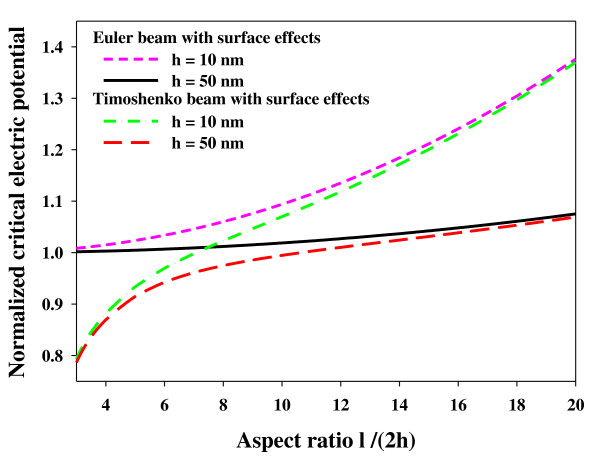
**Effect of nanowire height on normalized critical electric potential of the piezoelectric nanowires**.

Figure 3 illustrates the normalized critical electric potential versus the aspect ratio for various nanowire heights. In this diagram, the two modified Euler beam and Timoshenko beam models have been compared. The figure clearly shows that the normalized critical electric potential is size-dependent, and as the nanowire height gets smaller and tends to the nanometer size, the surface effects become more significant, and the difference increases between the classical theory and the modified theory in both the Euler and Timoshenko beam models. On the contrary, when the nanowire height increases, the curves converge each other, and the results approach those of the classical theories. This occurrence is the result of elimination of surface effects. Also, it can be realized that the surface and piezoelectric effects are more significant for a slender piezoelectric nanowire with a larger aspect ratio. Shear deformation reduces the normalized critical electric potential, and it has a greater influence on nanowires with smaller aspect ratios and improves the accuracy of the results compared to those obtained by the modified Euler beam theory.

In this study, the thickness of the surface layer has been disregarded because it is very small relative to the sizes of the nanowire's geometrical parameters. Also, in the present investigation, the assumption is that the deformation of the nanowire is small and that the resultant principle (the principle of superposition) can be used to sum up the tensions arising from the surface and piezoelectric effects; therefore, the effect of surface tension has been modeled as a curvature-dependent transverse loading, and the piezoelectric effect has been modeled as an induced axial force in the nanowire.

## Conclusion

In the present study, by applying the modified Timoshenko beam theory and considering the effects of surface, piezoelectricity, and shear deformation, the buckling behavior of piezoelectric nanowires was investigated. The obtained information can be used in the design and characterization of piezoelectric nanowire-based devices and instruments. The critical electric potential for the buckling of piezoelectric nanowires with the hinged-hinged boundary condition was derived analytically. The results show that, in addition to the surface effects, the shear deformation and piezoelectricity can effectively influence the buckling behavior of nanowires as well. Also, it was observed that, contrary to the surface effects, the shear deformation tends to reduce the critical electric potential and that it has a greater influence on stubby nanowires with smaller aspect ratios. Therefore, for smaller nanowire heights in the nanometer range, the effects of surface elasticity, piezoelectricity, and shear deformation should be taken into consideration so that more accurate results are obtained.

## Competing interests

The authors declare that they have no competing interests.

## Authors' contributions

ATS carried out the modified Timoshenko beam theory to analyze the buckling behavior of piezoelectric nanowires, participated in the design of the study, and performed the statistical analysis. GFW participated in the design of the study and checked the procedure of the solution and figures. MB participated in the sequence alignment and drafted the manuscript. All authors read and approved the final manuscript.
